# A Responsible Internet to Increase Trust in the Digital World

**DOI:** 10.1007/s10922-020-09564-7

**Published:** 2020-09-07

**Authors:** Cristian Hesselman, Paola Grosso, Ralph Holz, Fernando Kuipers, Janet Hui Xue, Mattijs Jonker, Joeri de Ruiter, Anna Sperotto, Roland van Rijswijk-Deij, Giovane C. M. Moura, Aiko Pras, Cees de Laat

**Affiliations:** 1 SIDN Labs, Arnhem, The Netherlands; 2 University of Twente, Enschede, The Netherlands; 3 University of Amsterdam, Amsterdam, The Netherlands; 4 Delft University of Technology, Delft, The Netherlands; 5 Wolfson College, University of Oxford, Oxford, UK; 6 NLnet Labs, Amsterdam, The Netherlands

**Keywords:** Trust, Digital sovereignty, Responsible Internet, Cybersecurity, Transparency, Accountability, Controllability

## Abstract

Policy makers in regions such as Europe are increasingly concerned about the trustworthiness and sovereignty of the foundations of their digital economy, because it often depends on systems operated or manufactured elsewhere. To help curb this problem, we propose the novel notion of a responsible Internet, which provides higher degrees of trust and sovereignty for critical service providers (e.g., power grids) and all kinds of other users by improving the transparency, accountability, and controllability of the Internet at the network-level. A responsible Internet accomplishes this through two new distributed and decentralized systems. The first is the Network Inspection Plane (NIP), which enables users to request measurement-based descriptions of the chains of network operators (e.g., ISPs and DNS and cloud providers) that handle their data flows or could potentially handle them, including the relationships between them and the properties of these operators. The second is the Network Control Plane (NCP), which allows users to specify how they expect the Internet infrastructure to handle their data (e.g., in terms of the security attributes that they expect chains of network operators to have) based on the insights they gained from the NIP. We discuss research directions and starting points to realize a responsible Internet by combining three currently largely disjoint research areas: large-scale measurements (for the NIP), open source-based programmable networks (for the NCP), and policy making (POL) based on the NIP and driving the NCP. We believe that a responsible Internet is the next stage in the evolution of the Internet and that the concept is useful for clean slate Internet systems as well.

## Introduction

The Internet has evolved from a local network for a small group of experts in the early 1970s to a global, continuously evolving infrastructure that supports a wide range of services and products that almost all businesses, governments, and citizens depend on today, even more so after the 2020 Covid-19 outbreak.

However, policy makers in regions such as Europe are increasingly concerned about the trustworthiness and sovereignty of the foundations of their digital economy [1–3], because it often depends on systems manufactured or operated elsewhere. For example, the European Union Agency for Network and Information Security (ENISA) recently articulated their concerns about Europe’s “digital sovereignty” [3]. They point out that the top 15 Internet companies in the world (e.g., Google, Facebook, and Alibaba) are either from the US or from China and not one of them from Europe. In addition, they highlight that European tech companies often get acquired by non-European companies (e.g., 53 were bought by US “tech titans” in 2011–2016). The risks they associate with these developments include European service providers and citizens losing control over their data and cybersecurity facilities, Europe no longer being able to meet their citizens’ norms and expectations, reduced competitive power, and drain of technical expertise [3].

While European policy makers are trying to curb this problem through new policy proposals (e.g., for Artificial Intelligence, 5G cellular networks, and the Internet of Things) [2] and initiatives such as a European federated cloud service [1], we observe that the Internet infrastructure has not received much attention yet in this context, except in an ad-hoc way, such as following reports on alleged security weaknesses in 5G equipment [4]. We believe this is a serious omission, because ultimately trust and sovereignty also require service providers and product manufacturers to be in control of their dependencies on the Internet infrastructure, specifically when it comes to security and resilience. This is particularly relevant for critical service providers (e.g., power grids, transportation systems, mobile networks, and manufacturing facilities), which have become increasingly dependent on computer networks [5]. For example, such providers want to know if they are routing their traffic through networks with equipment that might have backdoors [4].

To fill this void, we propose the novel notion of a responsible Internet, which aims to provide a higher degree of trust and sovereignty for critical service providers and all kinds of other users by making the Internet more transparent, accountable, and controllable at the network-level. This means users have (1) insight into the security attributes of chains of network operators (e.g., ISPs, data centers, and DNS operators) that carry or could potentially carry their data flows (transparency and accountability) and (2) are able to use these details to send their data flows through certain classes of network operators or request network operators to change the way they handle these flows (controllability), perhaps by changing the infrastructure itself with the help of policy makers. We believe that improving the Internet’s transparency, accountability, and controllability is key for users to trust the network and to be in control of their dependencies on the Internet infrastructure (and thus to be sovereign).

Without a responsible Internet, users will continue to be subjected to the current “black box Internet”, which has weak transparency, accountability, and controllability properties. For critical service providers, for example, this means they will mostly not know which network operators their services depend on and they will have little control over which classes of operators they would prefer to carry their traffic (e.g., based on these operators’ security posture). As a result, they may prefer to continue to use their own dedicated networks rather than a shared global Internet, which ultimately limits their flexibility and increases costs.

From a technical perspective, a responsible Internet introduces two new distributed and decentralized systems. The first is the Network Inspection Plane (NIP), which improves the Internet’s transparency and accountability by allowing a wide range of users to request measurement-based descriptions of the Internet infrastructure in terms of the chains of network operators that (potentially) handle their data flows, their interrelations, and their properties (e.g., their jurisdiction and if they use open source router software). The second is the Network Configuration Plane (NCP), which increases the Internet’s controllability by allowing users to specify how they expect the infrastructure to handle their data based on the NIP’s network descriptions.

We complement the NIP and NCP with a set of policies (POL) that help shape the network in the longer term, for instance to incentivize operators to use open source control and data plane software for security reasons or to join the NIP.

We make two contributions. The first is that to the best of our knowledge we are the first to define the concept of a responsible Internet, its properties (transparency, accountability, and controllability) and its key architectural components (NIP, NCP, and policies). Our second contribution is that we discuss research directions and starting points to realize a responsible Internet by combining three currently largely disjoint research areas: large-scale measurements for the NIP, open source-based programmable networks for the NCP, and policy making (POL) using insights gained from the NIP.

Our properties of a responsible Internet are inspired by those of responsible Artificial Intelligence (AI) [6]. The parallel with a responsible Internet is that policy makers worry about society’s level of control over AI systems as well [7] because they are black boxes similar to the Internet and because they also shape societies [6] (e.g., through AI-based parole and air pollution decisions [8]). The European Commission recently embraced a concept similar to responsible AI in their four principles for trustworthy AI [7], which they call the “explicability principle”.

Similar to responsible AI, a responsible Internet introduces a wide range of cross-disciplinary challenges, for instance in the fields of ethics, education, legislation, business models, and technology. While our paper focuses on technical challenges, our goal is to trigger and facilitate a wider, cross-discipline dialogue on a responsible Internet among stakeholders such as researchers, citizens, operators, and policy makers as well as to provide guidance for new research directions.

We think of a responsible Internet as the next stage in the evolution of the Internet, building on earlier and ongoing developments to increase the Internet infrastructure’s security (e.g., through technologies such as DNS-over-HTTPS, DNS security extensions, and a public key infrastructure for the routing system), resilience (e.g., through anycast), and privacy-friendliness (e.g., through DNS query name minimization). The concept can also be applied to “clean slate” infrastructures, such as based on SCION [9] or RINA [10].

We emphasize that our vision of a responsible Internet continues to follow the Internet’s open, bottom-up, and multi-stakeholder nature. Our notion of sovereignty is about service providers and individuals being in control of their dependencies on the Internet infrastructure and is explicitly not about creating government-controlled or even isolated national networks (cf. the “Beijing Internet” or the “Moscow Internet” [11]), nor is it about excluding technologies from specific regions [2].

In the rest of this paper, we first briefly outline scenarios to illustrate the added value of a responsible Internet for various types of users (Sect. 2). Next, we discuss the design of a responsible Internet (Sect. 3) and the research directions we identified for the NIP (Sect. 4), the NCP (Sect. 5), and the new policy mechanisms (POL) that a responsible Internet enables (Sect. 6). We continue with a discussion on the “Internet trust transition” that we think a responsible Internet facilitates (Sect. 7) and wrap up with an overview of related work (Sect. 8) and our conclusions (Sect. 9).

## Illustrative Examples

The purpose of a responsible Internet is to provide a higher degree of trust and sovereignty for a broad range of users. In this section, we illustrate what this entails using four simple scenarios: critical infrastructure providers (Sect. 2.1), policy makers (Sect. 2.2), network operators themselves (Sect. 2.3), and individuals (Sect. 2.4).

We envision that critical infrastructure providers, policy makers, and network operators will initially benefit the most from a responsible Internet. Individuals might benefit as well but will need novel user interfaces and additional guidance to enable them to navigate the network descriptions that a responsible Internet provides.

### Critical Infrastructure Operators

One of the key beneficiaries of a responsible Internet are critical infrastructure providers such as power grid operators and providers of intelligent urban transport systems. They benefit because a responsible Internet gives them more control over their dependencies on the network, which is essential to protect the security of their services and prevent large-scale incidents such as data breaches and safety risks for large groups of citizens.

As an example, consider a provider of a smart grid that sends flows of instructions to remote field stations to control power line switches [12]. In a responsible Internet, the grid provider can request the network to provide a description of how these flows travel through different networks, what type of equipment is used along the path, who operates the networks, and if any operations are outsourced to other networks (transparency and accountability).

In addition, the grid provider can request a responsible Internet to select an alternative network path [9] (controllability) based on the network descriptions it obtained earlier, for instance because they reveal that some network operators use equipment that might have software vulnerabilities (e.g., alleged back doors [4]). It can also use these descriptions to work with policy makers to request enduring changes through regulatory action (see Sect. 2.2).

### Enabling New Internet Policies

The network descriptions that a responsible Internet provides enable new types of policy making (developing the principles for a policy), policy mediation (translating the principles laid out in a policy to concrete, actionable steps), and policy enforcement (ascertaining that the steps are indeed implemented), which are three typical steps of policy development.

#### Policy Making

While classic policy making relies on consolidating input from stakeholders and taking interests and capabilities into account, a responsible Internet enables policy makers to take a more data-driven and proactive approach based on network descriptions. For example, a responsible Internet enables national policy makers to: (1) analyze risk areas in their local Internet infrastructure (e.g., concentrations of power or single points of failure [13]) based on historical data analysis; (2) infer models that help them play out realistic what-if scenarios; and (3) develop new regulatory strategies (e.g., to protect Europe’s digital sovereignty [2, 3]).

#### Policy Mediation

We expect that a responsible Internet will enable policy makers to act much faster upon emerging problems and risks, saving costs in litigation. For example, they could feed network descriptions into a platform that facilitates evidence-based feedback between parties. Also, critical infrastructure providers such as power grids and transportation systems can voice their concerns based on network descriptions obtained from a responsible Internet. For example, they can indicate that more network operators need to adhere to the “Mutually Agreed Norms for Routing Security” [14] to properly protect their services. In turn, policy makers can judge by the outcomes (e.g., tracked configuration changes) to determine whether further investigation or intervention is required.

#### Policy Enforcement

Policy makers benefit from network descriptions because they support data authenticity through cryptographic proof (see Sect. 4), which will help solve enforceable liability with respect to operators and third-party vendors (e.g., for operators of IoT services [15]). Regulators are able to understand in which society the operator is embedded (e.g., in terms of safety, privacy, freedom of speech, and laws for corporate and state surveillance [16]). The network descriptions present a useful interactive map for stakeholders who can efficiently identify the issues and the associated responsible parties, especially when Internet infrastructure is attacked. Law enforcement authorities can use the graph to map out key operators and identify areas for further investigation.

### Enabling Cross-Network Operator Incident Analysis

Another class of users of a responsible Internet are network operators themselves. For example, an operator that measures the properties of the DDoS attacks that it handles (e.g., Mirai-based DDoS attacks [17] or incidents similar to the 2015 DDoS attack on the DNS root [18]) can include the metadata of these datasets in network descriptions along with a usage license. The advantage is that it becomes much easier for other operators to find such datasets and the licenses to use the data [19]. This enables them to collaboratively combine and learn from each other’s measurements, which improves their collective incident response capabilities.

Ultimately, we envision that a responsible Internet enables the real-time sharing of measurements across network operators, allowing them to collaboratively fend off security incidents as they occur (e.g., by dynamically moving scrubbing functionality to a specific part of their network using Network Function Virtualization (NFV) [19]) or even proactively before they can cause real harm. Network operators could share the actual measurements in various ways, such as directly from their own servers or through a shared platform in which multiple operators upload their measurements (e.g., DDoS fingerprints [20, 21]).

### Giving Individuals more Insight in and Control over their Data

In the long term, we expect individuals to benefit from a responsible Internet as well. For example, people using video conferencing services (e.g., Zoom) could request a network description from a responsible Internet, which enables them to verify where their video flows end up and potentially change their endpoint to a data center in another region.

The Covid-19 pandemic of 2020 illustrated the relevance of this type of scenario. With lockdowns enacted in many countries, Zoom [22] emerged as one of video communication tools of choice. Confronted with a list of security issues, governments soon warned against using the software [23]. Among the cited concerns, the storage of cryptographic material in data centers outside “friendly” jurisdictions was considered problematic. Zoom reacted to this by allowing their customers to choose which data centers they wanted to connect to.

In a responsible Internet, these kinds of facilities would be built into the network infrastructure and thus be available for all applications, including Zoom.

## Designing a Responsible Internet

In this section, we present the outlines for the design of a responsible Internet, which builds on two new distributed and federated systems.

We discuss our notion of the qualifier “responsible” (Sect. 3.1), our proposed design goals (Sect. 3.2), our high-level architecture to realize these goals (Sect. 3.3), and the technical blueprint of network operators in a responsible Internet (Sect. 3.4).

This section is the starting point for the research directions that we foresee and elaborate on in Sects. 4 through 6.

### Origin and Meaning of “Responsible”

Our notion of a responsible Internet is inspired by work of the responsible Artificial Intelligence (AI) community, which focuses on giving people more insight in how AI systems reach decisions and why [8]. This is important because AI systems can have a profound impact on people’s lives. For example, there have been known cases in which AI systems incorrectly denied people parole or miscalculated air pollution levels [8]. Responsible AI extends the design and operation of AI systems with three design goals (transparency, accountability, and responsibility [6]) that help researchers, developers, and AI operators to consider the impact of their work “by design” (e.g., in terms of ethics) and not only focus on the predictive performance of their algorithms (e.g., in terms of accuracy).

The parallel with the Internet infrastructure is that the latter is a complex black box as well, that much of the focus of its development has been on its performance characteristics (e.g., response times, security, and resilience), and that it also may affect people’s lives in unpredictable ways, albeit more indirectly because it is a communications substrate that applications build upon [24]. For instance, a power grid operator may be reluctant to remotely control power lines at field stations over the Internet because it does not know the properties of the chain of network operators responsible for enabling the communication and cannot control them. Another example is that the opaqueness of the Internet infrastructure may lead to concentrations of power going unnoticed, resulting in individuals and businesses becoming overly dependent on large commercial players they have little influence over [13]. Finally, individuals typically do not know if their data passes through network operators they would not trust or that their employer would disallow for certain classified types of work.

The difference to AI systems is that the Internet has only one high-level task, which is to securely and reliably provide end-to-end communications. In addition, a large part of the Internet’s complexity stems from its decentralized architecture with distributed ownership and control [25], whereas in AI the complexity is in the decision making algorithms. Finally, the need for a responsible approach emerged relatively quickly in the field of AI, likely because the effects of its classification algorithms are more visible to users.

Similar to responsible AI, a responsible Internet extends the design of the Internet infrastructure with four design goals, which we discuss next.

### Design Goals

Inspired by responsible AI, we propose to update the design of the Internet so that its infrastructure becomes more transparent, accountable, and controllable at the network-level, which is how we define a responsible Internet. In addition to these three design goals, we also formulate a fourth one, which is that the functions that reinforce the Internet’s transparency, accountability, and controllability properties need to be usable by a wide range of end-users. Together, we think of our four design goals as extending the Internet’s original design goals, such as federating autonomously administered networks and survivability of failures [26].

#### Transparency

Transparency is the ability of a responsible Internet to describe its internal structure in terms of network operators, their properties (e.g., their jurisdiction and technical infrastructure), and their relations with other network operators. By network operator we refer to an administrative entity that operates a network, such as an access network, a transit network, a data center network, or a Content Distribution Network (CDN).

We distinguish two types of transparency:

1. Data transparency describes which network operators transport a particular data flow (e.g., instructions to configure a power grid’s field station) and how they process these flows. Data transparency typically requires network operators to track how they process data flows, for instance where a flow entered their network, which types of routers handled the flow, and where the flow left the network. Data transparency for instance enables power grid operators to track how flows of instructions reach field stations (see Sect. 2.1). A flexible implementation requires advanced network functions such as inband telemetry in open programmable networks (see Sect. 5).
2. Infrastructure transparency describes the infrastructure properties and relationships of network operators (e.g., their servers, routers, their geolocation, and the open source software they use), independent of specific data flows. Infrastructure transparency is based on self-declarations by network operators about their properties and relationships (e.g., the third parties they use) and on independent observers that map networks using large-scale measurement systems (see Sect. 4). Infrastructure transparency for instance enables policy makers to study the concentrations of power in an ecosystem such as the DNS (cf. Sect. 2.2).

Our notion of transparency is similar to that of responsible AI, which is about “the need to describe, inspect and reproduce the mechanisms through which AI systems make decisions and learn to adapt to their environment, and to the governance of the data used or created” [6]. Responsible AI does however not distinguish the concept of a flow, which is specific to computer networking.

#### Accountability

Similar to transparency, we consider two types of accountability:

1. Data accountability is about network operators explaining that they process specific data flows in a certain way, such as that they made certain routing decisions or that an intermediate network operator (e.g., a CDN) terminates TLS connections rather than the intended endpoint.
2. Infrastructure accountability is about network operators explaining that they designed their infrastructures in a certain way. These details can pertain to why they outsource parts of their operation (e.g., to flexibly provide DNS services in different parts of the world [27]) or why they use particular open source software.

Accountability requires actors to explicitly describe the norms (or ground rules) they use for decision-making. For example, network operators could indicate that they prefer to route their traffic through certain groups of operators, such as those that implement the MANRS rules for secure routing (e.g., to actively prevent the propagation of incorrect routing information) [14]. Similarly, a global cloud provider could inform its users that its default policy is to connect users to a local data center for performance reasons. As a result, Europeans traveling to the US would know that they will be using a US-based data center, which they may then ask the cloud provider to change (see controllability).

The norms are our equivalent of the “representation of the moral values and societal norms holding in the context of operation, which the agent uses for deliberation” in responsible AI [6].

A responsible Internet captures both transparency and accountability details in so-called network descriptions (see Sect. 4).

#### Controllability

Controllability is about the ability of users (e.g., critical infrastructure operators) to specify how they expect chains of network operators to handle their data based on descriptions of the Internet’s internal operation (see transparency and accountability). For example, a smart grid provider could use the Intent Definition Language [28] to indicate that it only wants to send instructions to remote field stations via certain classes of network operators, such as those that have certain security properties, are in particular jurisdictions, or that use verified open source stacks or certain types of routers.

Controllability requires new network functions such as path control based on multiple parameters, which we propose to implement using open source-based programmable networks (see Sect. 5).

A different form of controllability is through policy making, which operates at longer timescales and requires policy instruments (e.g., about allowed levels of outsourcing) rather than new network functions.

#### Usability

Usability is the ability of a responsible Internet to realize the other three design goals in an easy to use way for a wide range of users. This is important because users such as smart grid providers and policy makers will typically not be network experts and because the Internet infrastructure and the technologies it uses are complex, even for network experts. A responsible Internet therefore needs to provide transparency and accountability details about its internal workings at a high level of abstraction and in a machine-readable way so they can be interpreted by automated tools (e.g., to analyze network descriptions). Responsible AI offers some contrast in that usability is not an explicit design goal.

### High-level Architecture

**Fig. 1:**
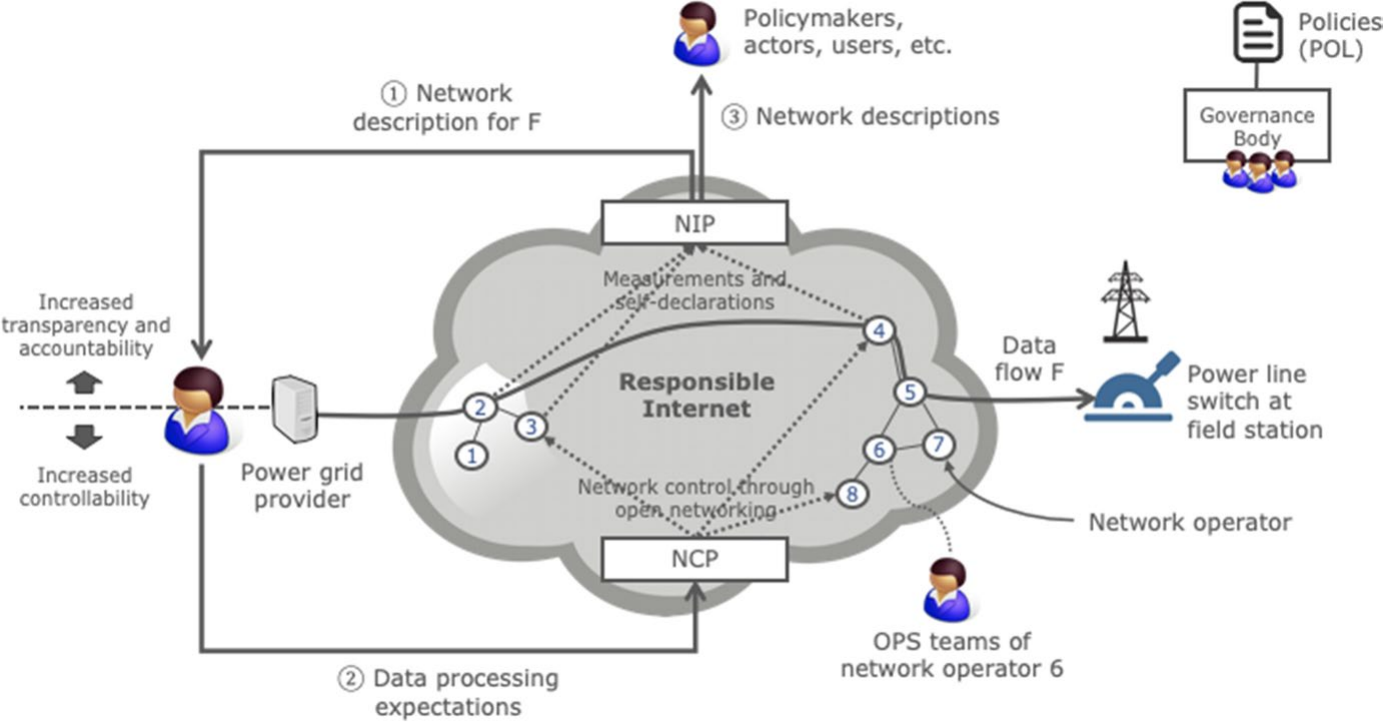
A responsible Internet’s key components (NIP, NCP, and policies) using a power grid provider as an example

Figure 1 shows our high-level architecture of a responsible Internet, using a power grid provider as an example user. Our architecture realizes the four design goals of Sect. 3.2 through two new distributed and decentralized systems (NIP and NCP), and a set of policies.

#### Network Inspection Plane (NIP)

The NIP improves the transparency and accountability properties of the Internet in a usable way. It allows users such as smart grid operators to query a responsible Internet for details about its internal operations in terms of network operators (interaction ①), which may include ISPs, DNS operators, and cloud providers. These network descriptions cover network operator properties such as their jurisdictions, technical infrastructure (e.g., routers, switches, servers, and their security posture), and relations with other network operators (e.g., outsourcing relations). Network descriptions can be based on a language such as the Network Description Language (NDL) [29, 30] or the recently proposed GAIA-X self-descriptions [31].

The added value of a network description is that it abstracts away from the underlying technical mechanisms to obtain the details about network operators (e.g., through large-scale measurement systems), which makes it useful for a wide range of non-expert users. For example, the power grid provider in Fig. 1 can use the NIP’s network descriptions to assess how instructions for remote power switches flow to field stations, while policy makers can use it to spot concentrations of power.

The network descriptions that the NIP returns can pertain to a specific data flow such as flow F in Fig. 1 (data transparency and accountability) or to the infrastructure irrespective of a particular flow (interaction ③). The latter type of information is relevant, for example, for policy makers (see Sect. 2.2).

The NIP populates network descriptions using various sources, including heterogeneous large-scale measurements from independent observers (e.g., using a system such as OpenINTEL for the DNS [32]) and open programmable telemetry functions in the infrastructure of network operators [33] (see Sect. 5). It also uses self-declarations from network operators (see Sect. 4), similar to the “self-descriptions” of GAIA-X operators [31] or the “cybersecurity labels” that large companies such as Deutsche Telekom, Ericsson, and Thales recently advocated for service providers and manufacturers [34].

The NIP provides mechanisms that enable users to verify the data source (e.g., similar to DNSSEC) that provides details about a particular network operator. This is important because it helps users trust the network descriptions that the NIP provides.

An equivalent of the NIP does not exist in the current Internet, because measurement systems are mostly not standardized and typically require scarce technical expertise of people such as network operator staff and security researchers.

#### Network Control Plane (NCP)

The NCP increases the controllability property of the Internet in a usable way. It is the counterpart of the NIP and enables users to specify how they expect chains of network operators to handle their data based on network descriptions (interaction ②). For example, the operator of the smart grid in Fig. 1 can use the NCP to indicate that instructions for power switches at remote field stations [12] should only pass through network operators in a certain jurisdiction or through network operators that have open sourced their data and control plane software. Similarly, customers of video services such as Zoom could use the NCP to select a video server on a data center in their own jurisdiction rather than a differently situated, default video server (see Sect. 2.4).

The NCP consists of a set of control and data plane services for open programmable network equipment that map users’ expectations to programmable network functions. It also contributes to the transparency property of a responsible Internet through open programmable telemetry functions (see Sect. 5).

The level of control that we envision for the NCP is much richer than in the current Internet, where control across networks is relatively one-dimensional.

#### Policy Framework (POL)

A responsible Internet also requires a set of policies that define the norms that network operators need to adhere to in terms of transparency, accountability, controllability, and usability. This includes auditing to check if requirements continue to be met. For example, the policy framework could define a basic level of responsibility that only requires network operators to publish rudimentary details such as their legal jurisdictions. Higher levels of responsibility could amount to network operators also sharing details on their relations with other operators, data plane telemetry, the geolocation of their servers, the source code of their data and control planes, and audits of data plane software.

The policy framework needs to be managed by a governance body, for which we envision a lightweight, multi-stakeholder model such as MANRS [14] for routing security. More “heavy weight” models are possible as well, such as a governing body that is part of ICANN, RIPE, or a national government.

For simplicity, we omitted the fourth interaction in Fig. 1, which is between policy makers (top) and the network operators. These interactions for instance involve the former incentivizing the latter to change their infrastructure to share details about their operation through the NIP (see Sect. 2.2), perhaps based on citizen-supplied network descriptions.

### Network Operator Architecture

**Fig. 2:**
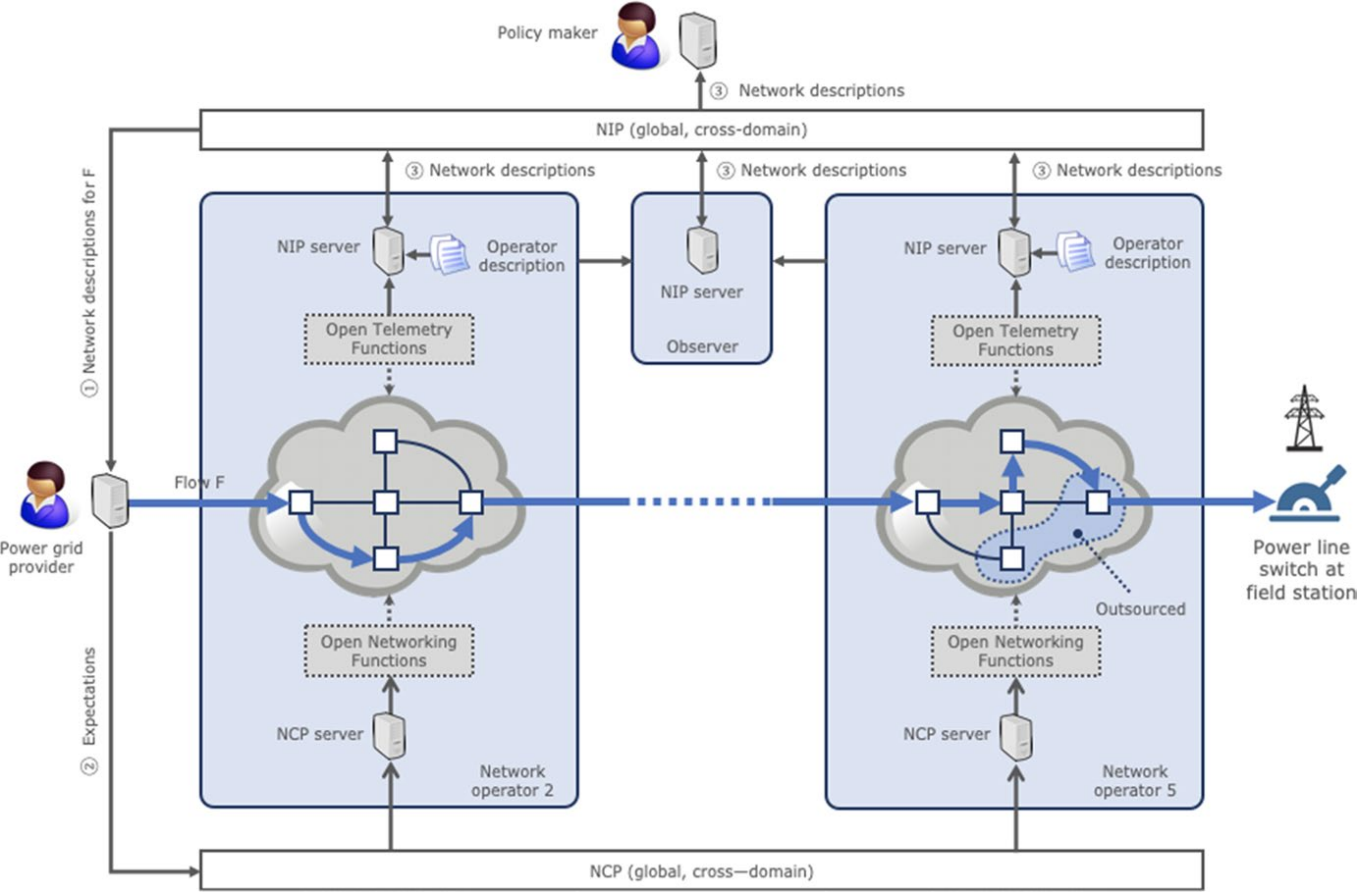
Architecture of a network operator in a responsible Internet

Figure 2 provides an overview of the architecture of a network operator in a responsible Internet, using Fig. 1 as an example. The numbers in Fig. 2 (①, ②, and ③) correspond to the interfaces in Fig. 1.

The main components of the operator architecture are:

- NIP server: locally stores the description of an operator and shares it with the global NIP. A NIP server also collects measurements from within the operator and acts as a NIP client to obtain descriptions of other operators from the global NIP. This includes enhancements of the operator’s own description with measurements from independent observers.
- Open telemetry functions: control and data plane functions for open programmable networks that collect fine-grained telemetry, such as network paths and routing table versions [33]. The telemetry functions provide input to the operator’s NIP server.
- NCP server: invokes networking functions that enable users to influence how a network operator processes their data flows (cf. Section 2.1) by calling programmable network functions.
- Open networking functions: predefined control and data plane modules that enable network operators to program their white box network equipment (routers, switches, etc.).

Observers implement a NIP server as well and use their “outside-in” measurements to enhance network operator descriptions.

Our key challenge is how to design, implement, operate, and evaluate the components of the architecture, which we will discuss in the next three sections.

## More Transparency and Accountability through the Network Inspection Plane

The Network Inspection Plane (see Sect. 3.3) increases the transparency and accountability of the Internet through high-level, measurement-based network descriptions of network operators (e.g., ISPs, DNS operators, and cloud operators), their relations, and their attributes. The NIP creates and populates these descriptions, which brings about many challenges.

We first discuss the concept of a network description in more detail (Sect. 4.1). Next, we present a first set of research challenges we identified to develop and evaluate the NIP (Sect. 4.2) and several measurement systems that can act as starting points to address these challenges (Sect. 4.3).

### Network Descriptions in More Detail

A network description is a machine-readable specification of the properties and relations of a group of interrelated network operators. A network description consists of network operator descriptions, which cover operator attributes such as:

- Services the operator provides (e.g., transit, DNS services, or CDN services)
- Types of relations with other network operators (e.g., delegation)
- Infrastructure (e.g., autonomous systems, router types, geolocations)
- Data and control plane details (e.g., software/hardware attributes)
- Data control capabilities (e.g., path control or geo-based end-point selection)
- Internet security incidents handled (e.g., domain or routing hijacks)
- Available measurements (e.g., DDoS traces, data plane telemetry)
- Norms used (e.g., MANRS or security audits)
- Applicable jurisdictions
- Support for security functions such as RPKI

**Fig. 3:**
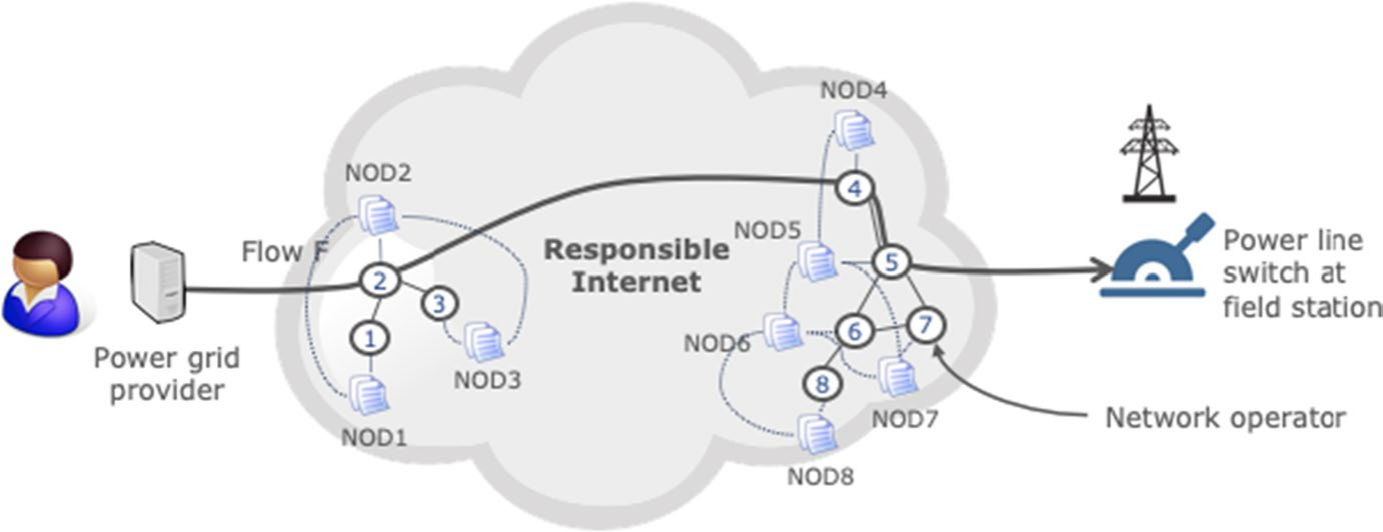
Example of a network description (based on Fig. 1)

Figure 3 shows a simple example based on Fig. 1, where the network description consists of network operator descriptions NOD1 through NOD8. The dashed lines between operator descriptions represent inter-operator relationships (e.g., between NOD1 and NOD2), while the dashed lines between a description and an operator indicates that the description pertains to that operator (e.g., NOD2 is the description of network operator 2).

The NIP populates a network operator description using two types of sources. The first are independent observers that carry out infrastructure measurements. For example, an observer like the OpenINTEL system [32] regularly obtains the DNS records of a wide range of TLDs, which enables it to map authoritative name servers to the networks where they reside and include these details in the descriptions of the TLD operators. Similarly, an observer such as the RIPE ATLAS measurement network can derive that a DNS operator outsources parts of its operations because its measurements reveal that clients end up at different anycasted DNS servers in different networks depending on the client’s geographic region.

The other source of information are network operators themselves. For example, they can add details on what other operators they peer with (cf. PeeringDB [35]), to which operators they outsource part of their operations, details about the types of equipment they use, their certification levels, and what measurements they carry out on their infrastructure that are available for collaborative incident analysis (see Sect. 2.3). The latter can for instance be based on the output of telemetry functions in open programmable networks [33] (see Sect. 5).

A network operator description captures these attributes at an abstract level (e.g., using the Network Description Language [29] or the recently proposed GAIA-X self-descriptions [31]), thus hiding the details of the various data sources from applications and making them easy to use.

### Challenges for the Research Community

We identified a first set of 7 high-level research challenges for the NIP, labeled NIP-RQ1 through NIP-RQ7.

#### NIP-RQ1: how do we model network operators?

The NIP needs an abstract data model to describe the properties of constellations of network operators like the one in Fig. 3. Sub-questions we identify are:

- How do we model a network operator? For example, what attributes do we need to capture (see Sect. 4.1), what are their semantics, and what is the appropriate breadth and depth of the model? This is important because network operators can have diverse properties. Also, should the scope of an operator description be all-encompassing or do we opt for a modular approach? For example, should we include details about physical Internet infrastructure as part of one monolithic model or as a separate module?
- How do we model relations between network operators? For example, they may delegate activities to other network operators, they may collaborate, but also commit to policies (e.g., MANRS for routing decisions). Accountability requires careful organization of information on dependencies, including unique identifiers for network operators but also standardized yet extensible relationship descriptions. All participating systems must be able to recognize these appropriately and, if necessary, update definitions. Accountability is also closely linked to verifiability, which we discuss in NIP-RQ3.
- How do we keep the data model forward-compatible and generic to also support clean slate Internet architectures such as SCION [9]?

#### NIP-RQ2: How do we Populate Network Descriptions?

Another challenge is how network operators and observers together populate network descriptions. This is a challenge because of the wide range of measurement systems that they use (e.g., passive and active systems), which are currently not standardized in terms of methodologies and output semantics. It is also a challenge because the measurement data will come from multiple vantage points (e.g., home networks or backend systems) and may conflict with each other [36]. Solving this challenge is important because the value of network descriptions is that they abstract away from measurement details so they become useful to a wide range of users.

A related question is how network operators update their operator description. For example, network operators should be able to automatically update their description through their NIP server (Fig. 2) when they change their infrastructure (e.g., when they outsource part of their operations to a third party). Also, operations teams should be able to verify their operator description before publishing it in the NIP.

Similar facilities to provide such details exist today, but in a scattered and unstructured way. For example, Internet operators provide assertions of network peerings through PeeringDB [35]. Similarly, some DNS operators blog about the software types [37] and third-party DNS operators [27] they use.

#### NIP-RQ3: How do we Validate the Authenticity of Network Descriptions?

The values in a network description (e.g., relations, policies, and available measurements) will typically originate from network operators and various independent observers (see NIP-RQ2), so we will need some way of validating their authenticity. In some cases, there will be a trust anchor, such as sources adding an RPKI-based signature to the value they provide. In other situations, we will need to revert to measurements from a variety of vantage points because there is no evident trust anchor.

One possible research direction is to augment the NIP with an append-only log that stores measurements similar to Certificate Transparency [38], which makes it possible to establish a causal chain of measurements documenting an event or configuration. The advantage is that these logs do not require universal verifiability and that attempts to tamper with results of previous measurements can be detected.

#### NIP-RQ4: How do we Design the NIP?

A key factor in the design of the NIP is the expected usage patterns of its two main services: (1) enabling users to look up the descriptions of groups of interrelated network operators and (2) allowing network operators and independent observers to update operator descriptions. For example, we could design the NIP as a hierarchical system similar to the DNS if its access pattern consists of relatively few updates of network descriptions and many lookups. If these patterns are more symmetrical, then a peer-to-peer design might be more appropriate.

Getting an indication of these usage patterns before building the system will require longitudinal measurements of how constellations of network operators and their attributes currently change over time, for instance in the DNS and in the routing system. A potential approach for the DNS is to study the changes in the DNS ecosystem, such as in a ccTLD.^1^

Another factor is the expressiveness of NIP queries, which should allow users such as the grid operator to search for network operators with certain properties (e.g., those with certain packet forwarding policies or security posture), amongst others.

Addressing this challenge requires a flexible system design, which is particularly important because the NIP is a cross-layer system: it provides a network-level service, but it populates network descriptions using measurements and declarations from different levels (e.g., network-level peerings as well as properties of equipment). This includes developing open standards that the NIP requires, for instance to facilitate interactions between NIP servers or express network descriptions (e.g., using the Network Description Language [29]).

#### NIP-RQ5: how can we validate the usefulness of the NIP?

Validating the added value of the NIP will require the development and evaluation of tools and algorithms that analyze network descriptions for various real-world use cases, such as those of Sect. 2. For example, these tools could query the NIP to calculate sector-specific “responsibility scores” of network operators based on raw network descriptions (e.g., for power grid providers or for citizens). Similarly, network operators could develop tools to automatically and collaboratively triangulate measurements of the same security incident (e.g., a DDoS attack) from multiple vantage points.

Validation also requires the development of target group-specific user interfaces (e.g., visualizations) that enable users such as power grid providers to easily and intuitively browse the NIP’s network descriptions and correctly interpret them for their specific use case. This will likely require new user interaction mechanisms, for instance to represent infrastructure concepts for users unfamiliar with networking and enable them to explore network descriptions at different levels of granularity.

The evaluation of the NIP will be a multi-disciplinary effort, requiring extensive consultation between domain experts, developers, and designers. This effort needs to be at the core of making a responsible Internet a reality.

#### NIP-RQ6: How do we Incentivize Network Operators to join the NIP?

Network operators will need an incentive to join a responsible Internet, because this will likely require significant investments from them, for instance in terms of technical facilities to join the NIP, adding support for open networking (see Sect. 5), and training their staff.

For the NIP, one possibility is to create a demand for the network descriptions that the NIP provides. A potential strategy to explore if such a demand exists is that a network operator interest group such as RIPE collaborates with “industry verticals” (e.g., critical service providers or consumers interest groups) to understand what kind of descriptions they would like to obtain from network operators. A small group of network operators could then set up a basic version of the NIP to pilot how this would work in practice, both for the network operators as well as for the users of the network descriptions. They could include their lessons learned in a set of implementation guidelines for other network operators to use, similar to the guidelines that MANRS provides [14].

As part of such a pilot, other types of users might develop new services based on network descriptions, such as a reputation system that calculates the “responsibility score” of a network operator. This would enable critical service providers and other types of users to easily discover network operators with “good” responsibility scores and prefer them to carry their data flows using services that the NCP provides (see Sect. 5). In this way, an initial small set of network operators may stimulate adoption towards a larger group because the transparency that the smaller group offers makes their services more appealing to users such as critical services providers (competitive advantage). A reputation system like this is similar to internet.nl, a site that enables users to check the security features of their ISP’s connection, amongst others.

NIP-RQ6 is related to the adoption of open networking and policy making, which we will discuss in Sects. 5 and 6, respectively.

#### NIP-RQ7: How to Balance Transparency and Security?

This last research question is perhaps the most important one for the NIP. Our notion of transparency and accountability implies that network operators share a certain amount of detail about their operations through network descriptions, but this may offer attackers quicker and more effective reconnaissance methods for possible targets. For example, sharing details on software versions might make a network operator more vulnerable to exploits. While a fast-paced patching cycle would solve this problem, this is not necessarily an option for everyone. For example, many organizations need to first test patches thoroughly for correct functionality before they deploy them in their production environment (e.g., in the financial industry).

We will thus need to look into the right level of detail to be included in network descriptions, which also ties into NIP-RQ1. A further direction to explore are ways to encode information in such a way that a malicious actor hardly profits, but a querying NIP user still receives useful results. There is precedence in the DNS: the DNSSEC NSEC3 record confirms the non-existence of a domain name while making it very hard for an attacker to enumerate those domains that do exist.

Solutions that address this research question will also require some form of standardization to achieve buy-in from network operators, for instance in the form of a cross-operator framework.

### NIP Starting Points

We discuss a few recent research results in the field of Internet measurements that can act as starting points for the challenges that we identified in Sect. 4.2. The measurement community developed these systems over the years because data availability and diversity is crucial to further our understanding of the Internet ecosystem (e.g., for DDoS characterization [39]). In this section, we discuss a few of these systems and how they could contribute to the NIP. We are involved in some of them.

#### OpenINTEL

OpenINTEL [32] has the long-term goal of collecting a daily comprehensive dataset on a significant part of the global domain namespace. It currently covers around 65% of the global namespace and collects over 3.7 billion data points every day.

The data collected by OpenINTEL can form the basis for an independent observer that covers large parts of the DNS and adds its measurements to network operator descriptions. It can also be used to perform a retroactive study of dependency developments in the DNS (cf. [40]), which provides details on inter-operator relations. Its reverse DNS dataset can augment transparency data on the IP layer.

#### Certificate Transparency (CT)

CT introduces logs to the certification process. These are neutral parties that can be run by anyone, although a spread across many different jurisdictions is desirable. Logs issue cryptographically verifiable receipts for every certificate they receive. Browsers can verify that the certificate they receive in a TLS connection has been correctly logged. CT’s notion of “transparency” has since been generalized in Google’s ongoing project Trillian [38].

The concept of transparency logs can be used for network descriptions to log measurements about operators or their relations from different sources. However, they may need to be scaled up because CT is designed for an ecosystem of just a few hundred actors (Certification Authorities). A small number of well-known logs is enough to enable this. A responsible Internet has operators orders of magnitudes larger (there are currently around 70.000 autonomous systems (AS-es) [41]), which usually are not aware of each other. Logs can in principle be run by any such operator, but an additional mechanism will be needed for the existence of logs to be communicated. Measurements are needed to validate information in the logs.

#### BGP Hijacking Event Analysis (HEAP)

HEAP [42] attempts to detect the cause of anomalies in the Internet’s routing system, such as legitimate inter-AS traffic engineering or attacks on an Autonomous System (AS). HEAP accomplishes this by combining a feed of “hijacking reports” with publicly available routing information and measurement data from Internet-wide scans. Routing hijacks are incidents in which an AS announces a route to an IP range that is a sub-prefix of a BGP announcement by a different AS. Such prefixes are generally globally accepted by all ASes and result in all traffic taking the new route instead of the old one. Ultimately, this is possible because BGP does not offer any security itself (RPKI has some deployment but is not widely used to filter routes).

HEAP uses descriptions taken from Regional Internet Registries (RIRs) to reason about legitimate business relationships between ASes. For example, RIRs such as RIPE store relationships of the form “maintained by” between ASes. This indicates that an incident is most likely benign as one AS has informally described an outsourcing of responsibility to another AS. The transparency in the responsible Internet we envisage would be a superset of such descriptions.

#### MADDVIPR

MADDVIPR^2^ (Mapping DNS DDoS Vulnerabilities to Improve Protection and Prevention) aims at comprehensively analyzing the DDoS landscape targeting the DNS (e.g., in terms of characteristics of DDoS traffic). The project stems from the observation that DDoS attacks on the DNS can have devastating effects [17, 40], as it effectively leads directly to loss of connectivity for end users and services.

MADDVIPR can contribute to the creation of network descriptions because it is able to shed light on the DDoS weak points of the DNS landscape. For example, it is able to map single points of failure in the global DNS and vulnerabilities [43] in DNS deployment that DDoS attacks can exploit. MADDVIPR also aims at mapping DNS DDoS “hotspots”, in terms of attackers, attacks and targets, which is relevant for network descriptions as well.

## More Internet Controllability through the Network Configuration Plane

The NCP (see Fig. 1) consists of a set of control and data plane services for open programmable network equipment, which network operators use for two purposes: to enable users such as grid operators to express a limited number of high-level data processing preferences (controllability) and to provide new data plane telemetry functions (transparency and accountability).

We envision that open networking (networking based on open source software and open programmable networks) will play an important role to realize a responsible Internet, but we identify a number of open problems.

We discuss our notion of open networking (Sect. 5.1), our research challenges for the NCP (Sect. 5.2), and starting points to address them (Sect. 5.3).

### Open Networking and a Responsible Internet

We define open networking as network equipment that uses open source software (e.g., based on OpenSourceNetworking^3^) and open hardware modules (e.g., based on the Open Compute Project^4^). Open networking is important for a responsible Internet because it enables users such as the grid operator of Fig. 1 to verify the security of these modules, which enables higher levels of trust and sovereignty. Network operators in a responsible Internet share details about the software and hardware they use through network descriptions (see Sect. 4) and their local NIP server (Fig. 2).

Open networking requires network equipment that can be programmed. Until a few years ago, networking hardware (routers/switches) were the proverbial black boxes that came with vendor-specific software that could be configured to some degree but could not be changed (re-programmed). Moreover, the hardware integrated both the equipment’s control plane (protocols and algorithms needed to make routing decisions) and its data plane (forwarding of packets). This hampered innovation, as adopting any new protocol basically required purchasing a new device.

While programmable networks have been studied since the 1990s [44], Software-defined Networking (SDN) [45] introduced a new type of networking hardware that separated the control and data plane functions, allowing the control logic to be programmed (by the user) in software and the corresponding rules to be installed in the data plane. In addition, new types of programmable network hardware allow engineers to flexibly develop custom hardware-based packet processors for the data plane, for instance to extend IPv6 packets with custom headers [33, 46] or implement a “clean slate” protocol such as SCION [9]. A popular language that supports this kind of programmability is P4 [47].

From a functional perspective, control and data plane programmability is important for a responsible Internet because it enables network operators to develop and standardize new network functions that allow users such as grid operators to indicate their data processing preferences for chains of operators (see Sect. 2.1), thus increasing the controllability of the Internet infrastructure. Operators enable users to express these preferences in a language such as the Intent Definition Language and implement them on programmable hardware (e.g., using P4) [28].

Programmable networks are also important for the NIP (Sect. 4) because they enable operators to add fine-grained telemetry from the data plane to network operator descriptions. Finally, programmable networks enable operators to manage their networks in new ways, such as through custom traffic management functions to handle DDoS attacks.

### Challenges for the Research Community

We identify 8 open networking-related research challenges to realize the NCP, which complement the NIP challenges that we discussed in Sect. 4.2.

Our research questions cover aspects related to exposure of telemetry data (NCP-RQ1 and NCP-RQ2), the security implications of such transparency (NCP-RQ3 and NCP-RQ4), the effects on users of open networking techniques (NCP-RQ5 and NCP-RQ6), and future extensions (NCP-RQ7 and NCP-RQ8).

#### NCP-RQ1: What Open Telemetry Measurements are Useful for Network Descriptions?

This is important because open networking and in particular programmable data planes allow for an unprecedented level of telemetry [33, 48]. Examples of measurements include the path that a flow takes through an operator’s infrastructure, the version of the routing table that each router uses to make routing decisions, the source and type of open source software used, and the operations that a router applies to the packets in a flow (e.g., forward, decrypt, sinkhole). The NIP can for instance use these details to enable data transparency and accountability (see Sect. 3.2).

A related research question is the appropriate granularity and frequency of the measurements, which is an important consideration for routers and a network operator’s NIP server (see Fig. 2). For example, the sampling frequency of the telemetry system needs to be such that it still allows for line speed forwarding of large numbers of flows.

Another key question is how to enable users to verify the authenticity of data plane measurements, which is related to NIP-RQ3 (Sect. 4.2).

#### NCP-RQ2: How do we get Data Plane Measurements into Network Descriptions?

This is important for data transparency, so users get insight into which operators processes their data flows. One possible solution is that routers include measurements in the packets themselves (e.g., using IPv6 extension headers) and forward it to the next hop (“packet forwarding state” [9]). For example, the border router of operator 2 (Fig. 2) could add its measurements for flow F to outgoing packets in extension headers so that operator 5 can upload them into the NIP through its NIP server for the whole operator chain. While this could be a feasible approach (SCION’s path transparency functions work similarly [9]), its downside is that it increases message size which is a disadvantage on wireless networks, which are typically bandwidth-constrained.

An alternative is that each network operator retrieves the data plane telemetry from their routers and adds it to network descriptions through their local NIP server. The potential downside of this approach is that it requires extra state in the network operator’s control plane, which increases its complexity.

#### NCP-RQ3: How to Protect the Integrity of Open Source Data and Control Plane Software?

Similar to the NIP (see NIP-RQ7), a major challenge for the NCP is how to balance the openness of data plane and control plane software and their security in terms of vulnerabilities that can be exploited.

One potential direction is to enhance network control programs with run-time attestation of these programs’ binaries [49], which enables network operators to verify the integrity of execution paths in the code and that they have not been changed by attackers. Similarly, network operators can also use static attestation to check the integrity of binaries by computing a hash over it at boot time and making them available for lookup [49], for instance as part of a network description.

Another way to protect the integrity of open data and control plane programs is through auditing. For example, the set of policies in the overall architecture of Fig. 1 could not only define responsibility levels but could also set requirements for open data and control plane software that network operators need to adhere to. Ultimately, such auditing mechanisms could become part of operational security best practices such as ISO270001.

#### NCP-RQ4: How do we Promote Adoption of Open Networking Systems?

Similar to network operators joining the NIP (see NIP-RQ6), another challenge is to develop mechanisms and incentives to stimulate the adoption of open networking so the concept of a responsible Internet can be rolled out gradually. Without open networking, a responsible Internet would require a complete overhaul of all Internet equipment and software, which would be virtually impossible.

A major challenge is to enable network operators to understand what joining a responsible Internet means for their business model. For example, they might need to redimension their infrastructure because their “responsibility score” results in users sending additional traffic through their network. This may be an advantage if the users are paying customers, but it might be a disadvantage if they are some other operator’s customer. In this case, a responsible Internet will likely also have an impact on the business relations between network operators.

As part of developing a business case for open networking, network operators will also need to understand what investments they will need to make to change their operations (e.g., in terms of new equipment, educating staff members, and operational costs). Early adopters of the concept could include such lessons learned in a set of implementation guidelines (cf. NIP-RQ4), which the governing body (see Sect. 3.2) could further promote.

NCP-RQ4 is related to policy making, which we will discuss in Sect. 6.

#### NCP-RQ5: How can Open Networking take Advantage of the Additional Insights that Network Descriptions Offer?

Open networking allows for a large degree of flexibility, which can be driven by the details that the NIP provides. The challenge for network operators is how to map network descriptions to the network control programs of the NCP.

For example, open programmable devices allow for adaptive rerouting of data flows among various public and private entities for crowd management applications. These changes can be driven by devices dynamically analyzing network descriptions to find weaknesses in the network that are a problem for this specific type of application and that they can therefore solve more effectively.

Similarly, network operators can proactively change their network (e.g., using VNF for fine-grained adaptations) because network descriptions of operators [20, 50] provide them with a more comprehensive view on what is going on in the network (cf. Sect. 2.3). The decision where to place a network function [51] will play a big role in how network operators perform in a responsible Internet. The ability for operators to take autonomous decisions in response to security incidents [19] will require further investigation.

#### NCP-RQ6: What will be the Effect of NCP on Users?

NCP users will likely also require novel interaction concepts (cf. NIP-RQ5), specifically to express their preferences on how chains of network operators should handle their data flows. For example, users such as grid operators might need an extension of their control room clients to include such network controls.

#### NCP-RQ7: How can we Leverage Open Networking to Evaluate and Extend the Concept of a Responsible Internet for Other Internet Architectures?

Programmable networking systems enable researchers to more quickly experiment with non-IP architectures, such as SCION [9], RINA [10], and NDN [52]. An open challenge is how to specialize the concept of a responsible Internet for these architectures. With the uptake of languages such as P4, we expect an increase in new architectures that will coexist with the current IP-based Internet. Recent efforts such as [53] identify the emergence of network virtualization and network programmability as the components that will allow the development of future Internet infrastructures. We build our work on the same insight.

#### NCP-RQ8: What are the Scalability Limits of the Information Exchange Required by the NCP?

A key challenge is how to scale a responsible Internet to large numbers of users. This will for instance require a careful design of the mechanisms that maintain the additional state that a responsible Internet requires. For example, open telemetry will enable operators to summarize how they processed a user’s data flow but sharing these details as packet forwarding state across operators (see NCP-RQ2) might result in too much overhead at the IP-level.

Similarly, users might need to express their data processing preferences at different levels of granularity in order for the system to scale. For example, users wanting to reroute their flows through different network operators might need to choose from several predefined paths like in SCION path control [9] because full per-flow routing will not scale.

### NCP Starting Points

We discuss a few recent research results in the field of open networking that can act as starting points for the NCP, which we are involved in.

#### The Netherlands’ National P4 Testbed

**Fig. 4:**
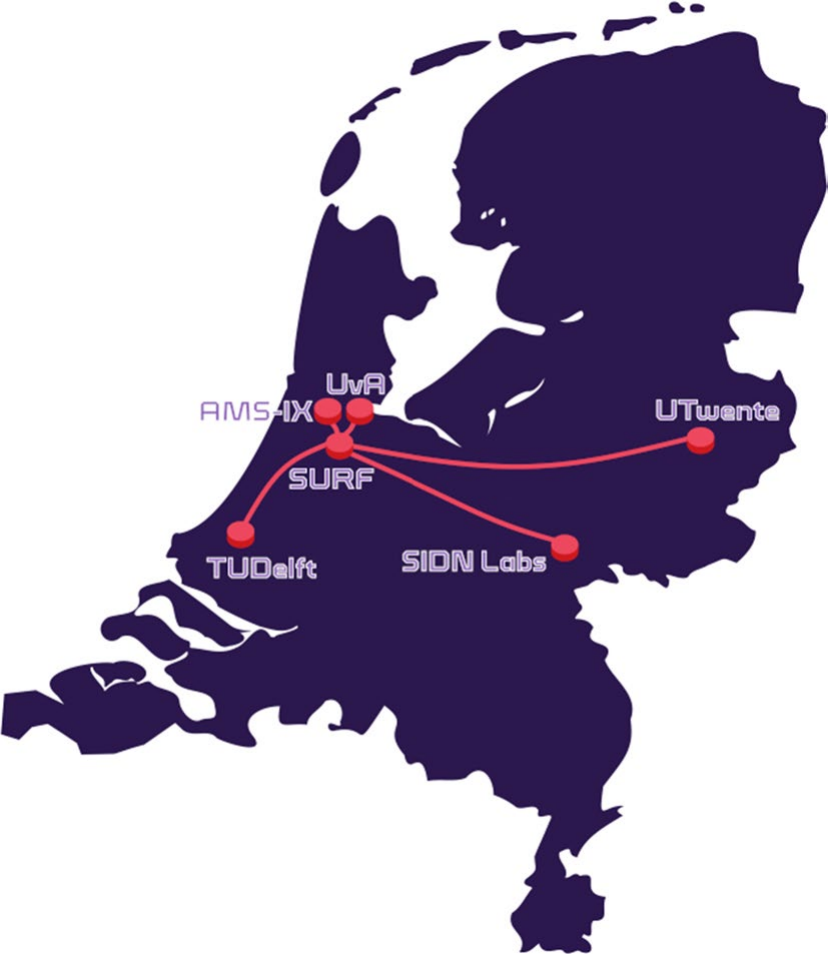
Netherlands’ national P4 testbed (March 2020)

A consortium of 3 Dutch universities and 5 companies (e.g., the Dutch national research and education network and two Internet exchange points) called “2STiC”^5^ recently set up the first national multi-domain P4-programmable network in the Netherlands (see Fig. 4). The testbed uses switches and NICs that can be programmed through P4. It consists of six different sites interconnected by a star-shaped optical network, which can be configured to use different topologies.

The programmability of the 2STiC network makes it very suitable to experiment with novel network functions, such as the ones that the NCP needs.

#### SCION-in-P4

“SCION-in-P4” is a P4 implementation of the SCION data plane protocol. SCION (Scalability, Control, and Isolation on Next-Generation Networks) [9] is a clean slate internet architecture that, for instance, aims to enable users to control the inter-domain routes their traffic takes (i.e., which autonomous systems their traffic passes through). We are currently testing SCION-in-P4 on (parts of) the 2STiC testbed of Fig. 4.

This work is relevant to extend the concept of a responsible Internet to other types of network infrastructures.

#### Data Plane Telemetry

We developed and experimented with several P4 telemetry mechanisms. For example, Sequential Zeroing [54] is a heavy-hitter (i.e., big flow) detection solution for P4-programmable hardware. It operates at line rate, which leads to new types of optimization problems because P4 programs need to adhere to the stringent memory access rates of programmable hardware.

Also, in [55] we enable programmable switches to (1) track processing and queuing delays of latency-critical flows and (2) react immediately in the data plane to congestion by rerouting the affected flows. Another example is our in-band telemetry implementation for the 2STiC testbed (Fig. 4), which appends node identifiers to IPv6 extension headers [48] so that destination nodes can extract the full path a packet took from these headers.

These data plane telemetry mechanisms are a starting point for the NCP’s open telemetry functions, for instance to add flow-specific details to network descriptions.

#### Network Slicing

We also used P4-programmable switches to dynamically create, discard and switch network slices (i.e., reserved resources, with known latency characteristics, dedicated to a specific type of application) [56]. This approach demonstrates how Quality-of-Service (QoS) can be attained for dynamic applications that require stringent latency constraints, such as remote surgery, which is relevant for the NCP.

While this network slicing approach takes its decisions based on real-time measurements from the data plane, it could also be extended to incorporate more information from the NIP.

#### P4 Code Generation

We also experimented with the automatic generation of P4 code (based on intents) [28, 57], thus providing first steps towards networks that can adapt themselves with only a few high-level commands from the users or operators (self-programming networks).

This work is relevant for a responsible Internet to manage the quality of the P4 code that the NCP uses and to develop a (standardized) repository of P4 network control software that network operators can choose from.

## Policy Mechanisms

A responsible Internet not only introduces technical challenges (see Sects. 4 and 5), but policy challenges as well, such as how a responsible Internet enables better policies and how to incentivize network operators to join the NIP and adopt the NCP.

We first provide a short background on how policies are typically being developed (Sect. 6.1) and then discuss the research challenges we identify (Sect. 6.2). We conclude with an overview of policy forums that are potential candidates to address these challenges (Sect. 6.3).

### Policy Development Background

Policies are made for users with very diverse technical knowledge and skill sets: from savvy, advanced users to late adopters. The policy community needs to understand the information available about a responsible Internet at each stage: policy making, policy mediation, and policy enforcement (see Sect. 2.2).

A common deficiency in governing the Internet is that policy makers, especially regulators, have difficulties following the pace at which technical developments occur. For example, the recent (and possibly short-lived) explosion of cryptocurrencies as “regular” forms of payment, or smart contracts as semi-autonomous, self-executing code, led to enormous uncertainties in terms of how to regulate cryptocurrency exchanges or code in smart contracts [58].

Other research on multi-stakeholder governance approaches also highlighted the increasing importance of the private sector in Internet policy, visible for example in standardization and data protection regulation (e.g., private companies such as Google and Facebook affecting legislation drafts at an early stage) and proposals for public–private actions to fight botnets [59].

### Challenges for the Policy Community

We identified four policy-related research questions, labeled POL-RQ1 through POL-RQ4.

#### POL-RQ1: How to Incentivize (Large) Network Operators to join the NIP and Adopt the NCP?

A responsible Internet will need to grow incrementally, like the Internet itself did. However, it might not result from market pressure alone, which is unlike communications-driven properties such as lower latencies and increased bandwidths that improve applications such as video conferencing [60].

As a result, an important challenge to deploy a responsible Internet is to develop incentives that stimulate a few “first movers” to join the NIP and adopt the NCP. This is a challenge because it requires network operators to invest in changing their infrastructure, for instance to switch to open programmable networks and train their staff (see NIP-RQ6 and NCP-RQ4). Policies that provide these incentives might range from voluntary similar to MANRS [14] to mandated by national regulators.

Another challenge is how to give network operators equally fair possibilities to participate in a responsible Internet. This is important to stimulate competition, which is a driving force in innovation and the inclusion of more diverse network operators should help a future responsible Internet thrive as well. A related challenge is how to get support from existing standardization bodies to encourage more operators to develop and adopt the NIP and the NCP. A potential hurdle to take is that large corporations often play important roles in numerous standardization bodies [61] (e.g., W3C, IEEE, and the IETF).

Another dimension is how to incentivize large organizations (e.g., large ISPs or content providers) to join a responsible Internet. In such cases, there is a risk of “regulatory capture” [62], which means that a few large dominant Internet actors use their economic power to shape potential legislation aimed at stimulating a responsible Internet in favor of their own interests [63]. This type of risk is real and has been described for diverse scenarios of today, in particular cloud services, modern AI, and data-driven businesses.

#### POL-RQ2: How do we Ensure that a Responsible Internet Represents the Interests of the Public, Particularly in Critical Infrastructures?

International governmental organizations often advocate values that they would like to see reflected in the development of a future Internet. For example, the United Nations highlights nine values: inclusiveness, respect, human-centeredness, human flourishing, transparency, collaboration, accessibility, sustainability, and harmony [64]. The EU envisions the next-generation Internet as more human-centric, supporting openness, decentralization, inclusiveness, and the protection of privacy, while giving control back to the end-users, in particular with respect to their data [65]. The EC also articulated these kinds of values for AI [7].

It is significant to continue to research how these values are reflected in critical infrastructures (e.g., energy grids or transportation systems) that use a responsible Internet in countries that favor diverging values or that prioritize them differently. For example, how should critical infrastructure operators across countries incorporate network descriptions (see Sect. 4) in their services to reflect the above-mentioned common values?

#### POL-RQ3: How can the International Policy Community Collaborate Towards a Global Responsible Internet at a Time of Fragmentation?

Internet fragmentation, sometimes referred to as “Balkanization”, refers to nation states applying territorial control to their networks. This development has been long debated [66, 67] and it is well-known that several countries contribute to this process by deploying topic and domain-based filtering at large scale.

Internet fragmentation along territorial borders forms a major challenge for a global responsible Internet, for instance to incentivize national policy makers with varying expectations regarding fragmentation to collaborate. At the EU level, it will be important to examine how member states will utilize transparency features such as network descriptions in an effort to harmonize regulation and strengthen the Digital Single Market (DSM).

#### POL-RQ4: How do we Adequately Translate Policy to Different Target Groups (e.g., those in Sect. 2) to Ensure the Values and Function of the Future Internet?

To implement new policies on a largely privately owned and operated Internet, policy makers also need to help service providers translate the values of POL-RQ2 to responsibility profiles for network operators. For example, service providers and other users need to be able to make sense of the new kinds of details that a responsible Internet provides and make the right decisions in their own context. This particularly applies to individuals that are marginalized in the social-economic spectrum.

We expect this kind of research to grow into several smaller research areas that rely on empirical analysis and investigate actual implementation and impact. This breakdown is important because of the complexity of the work. For example, the EU now has complex policies related to their digital agenda [68] and it is unclear whether these policies are sufficient to address the issues raised in the context of a responsible Internet (e.g., in sectors such as energy, finance, and medical care).

It will also be important to evaluate how responsible Internet technologies are actually used when different social groups participate (e.g., policy makers and regulators, service providers of critical infrastructures, and individuals). A strategic approach is also required to initiate public and private partnerships and cross-disciplinary research to understand how a responsible Internet is used in different social-legal-cultural contexts.

### Policy Starting Points

Our research questions illustrate that a concerted effort will be necessary to make a responsible Internet a reality. No single country or organization is able to determine future standards alone, at least if they are to be used by a majority of service providers, device manufacturers, and operators. Fortunately, there are a number of forums and consortia that may serve as good starting places and that already have similar items on their agendas.

#### Internet Governance Forum (IGF)

The IGF is a venue to increase awareness of Internet governance, foster conversation, and educate the market.

The themes of a responsible Internet are represented across three of the IGF’s current four core policy agendas: an #OnlinePeaceFramework, a #DigitalMarshallPlan, and #OnlineRights4all. The fourth policy agenda, #ResponsibleAIStewardship, is the pendant of the responsible Internet in AI [69].

#### High-Level Panel on Digital Cooperation

The United Nations High-Level Panel on Digital Cooperation also highlighted better ways of digital governance in its report “The Age of digital interdependence” [64]. The report proposed three mechanisms to support an inclusive approach for global collaboration on Internet governance.

- Internet Governance Forum Plus (IGF+) aims to bring more representatives together and promote more actionable outcomes from discussions.
- The Distributed Co-Governance Architecture (COGOV) is dedicated to building a network to design and promote digital norms which policy makers can use as a blueprint to develop their policy, regulation, and laws.
- The Digital Commons Architecture (DCA) works on key issues around social harms to promote established digital commons.

These policy mechanisms can be utilized to foster conversations with a wide range of users and to initiate educational programs to increase awareness of a responsible Internet. These dialogues lead to strong international discourse and help increase users’ awareness of their rights in the digital space.

#### Council of Europe’s Strategy of Internet Governance

At a regional level, the Council of Europe’s (CoE) strategy of Internet Governance concentrated activities in three areas: building democracy online, ensuring online safety and security for all, and respecting and protecting the human rights of everyone in the digital world [70]. Along this strategy, the CoE initiated partnership agreements with eight leading Internet companies including Apple, Deutsche Telekom, Facebook, Google, Microsoft, Kaspersky Lab, Orange, Telefonica, and Cloudflare, as well as six international associations. The goal was to tackle issues including bioethics, data protection, disinformation, and cybercrime.

The starting approach could be, for example, to join the partnership and collaborate with the CoE to address high-priority issues concerning safeguards for internet critical infrastructure.

## Internet Trust Transition

We believe a responsible Internet enables a global Internet that is trusted by billions of non-expert users with widely varying norms and expectations [16]. This is a move away from the Internet’s original 1960s–1970s trust model, which revolved around a then-local Internet and a relatively small and homogenous group of expert users trusting each other (personal trust [71]) [26].

**Fig. 5:**
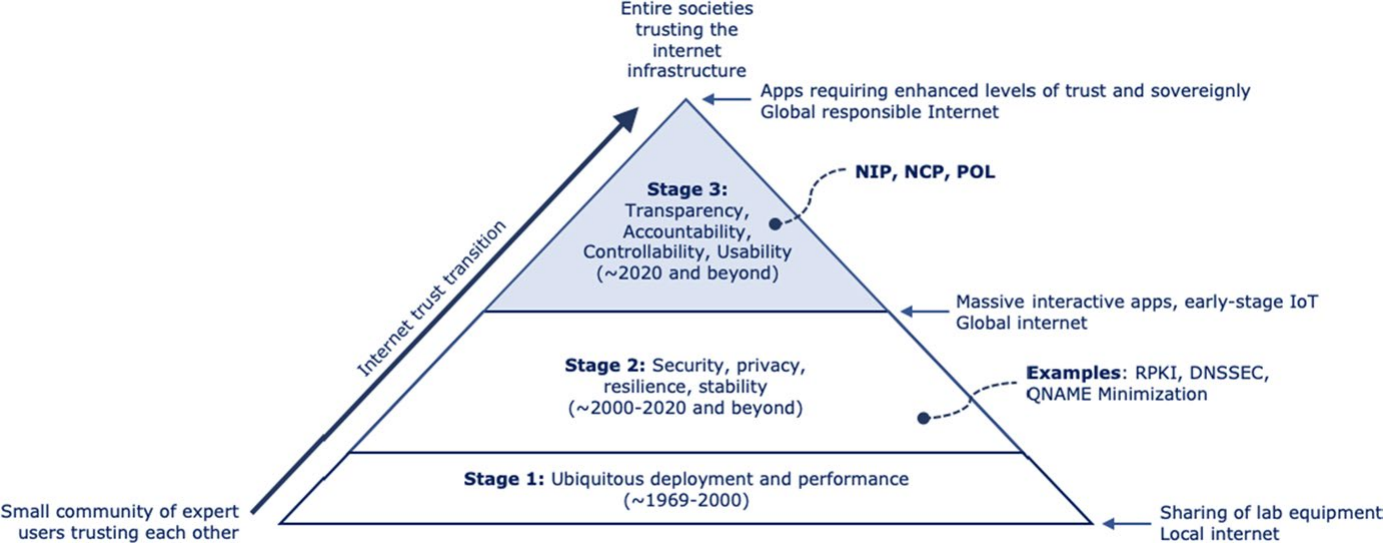
Internet trust transition (left arrow going up)

We call this change the Internet trust transition. Figure 5 shows that we think of it as a movement across the layers of a Maslow-like “pyramid of Internet needs”. A responsible Internet is at the top of the pyramid and builds on the advances achieved in roughly the previous two decades (the second stage of Fig. 5), in which the research and operational communities significantly increased (and are still increasing) the security, stability, and privacy-friendliness of the Internet. Examples of technologies that they developed, standardized, and deployed for this purpose include certificate automation (e.g., through Let’s Encrypt), certificate transparency [38], DNS security and privacy (e.g., through DNS security extensions and query name minimization), and routing security (e.g., using the Resource Public Key Infrastructure). The lower layer of the pyramid represents the period from the inception of the Internet in the late 1960s/early 1970s to the end of the 20th century, which focused on sufficiently maturing the Internet as a technology and getting it ubiquitously deployed [72].

A secondary transition that we believe a responsible internet needs to facilitate is what we call the value transition: from a relatively homogeneous set of norms and expectations of the community that governed the design and operation of the Internet in the early days (stage 1 in Fig. 5), to a broader and much more heterogeneous set enhanced with the norms, laws, and expectations of the different societies in which the Internet is embedded today (stages 2 and 3) [16]. The need for such a transition for the Internet is exemplified by recent work in Europe, where they are already actively seeking to align technological developments with European norms and expectations, for instance in cybersecurity [3] and AI [7].

We believe a responsible Internet enables this transition because it adds transparency and accountability as first class citizens to the Internet, which are important values in many societies as well as in Internet governance bodies (e.g., at ICANN and the IETF). It does mean, however, that the Internet increasingly embodies human values and that it becomes even less “value free”, which is a well-known tussle [16].

## Related Work

To the best of our knowledge, we are the first to propose and define the concept of a responsible Internet and to provide research directions for it. Our work is also unique because we join three existing but largely disjoint research areas: large-scale measurements (through the NIP, see Fig. 1), open networking (through the NCP), and policy making (using the NIP). The related work we did find addresses isolated aspects of our proposal.

### Internet Evolution

The need to evolve the Internet architecture has been forefront in the networking research community for a long time: from seminal work such as that of Chowdhury et al. [73] where network virtualization was proposed as solution to the Internet ossification, to a very recent proposal such as Trotsky by McCauley [53] that puts the focus on the use of programmability to allow multiple Internet architectures to coexist. Our work, however, goes one step further because we also include the policy perspective, which is unlike these efforts that primarily have a technological focus.

### GAIA-X

GAIA-X [31] is an ongoing project to create a cloud infrastructure and data ecosystem to improve Europe’s data sovereignty.

Similar to our responsible Internet, GAIA-X also advocates “responsible” design goals such as transparency and accountability. Another similarity is GAIA-X’s concept of self-descriptions, which is similar to our network operator descriptions. Nodes (an abstract term that can refer to elements such as data centers, network services, and virtual machines) can self-describe their characteristics, which are meant as inputs for users (consumers and providers) to select the level of data security they need. Self-descriptions can be certified by trusted parties and may refer to self-descriptions of other GAIA-X actors, effectively creating a self-description graph.

The key differences with or work are that we focus on the network-level. For example, GAIA-X currently allows for the self-description of network operators that cloud operators directly connect to (e.g., PoPs and transit providers), but unlike our work they do not consider transparency of the end-to-end communications path, nor do they outline the corresponding measurement systems. Another difference is that they do not consider an equivalent of our NCP. We thus consider our work complementary to GAIA-X.

### Open Internet Order

Lehr et al. [74] discuss the FCC’s Open Internet Order (OIO in short, superseded by the FCC’s Restoring Internet Freedom Order^6^ on June 11, 2018), which aimed to promote open broadband Internet access. Lehr et al. argue that Internet information disclosure and transparency (D&T) are important for different actors, such as ISPs, regulators, and customers. Their D&T policies involve information disclosure along 5 dimensions, such as why disclosure is needed and what data needs to be disclosed. From a network perspective, D&T may pertain to disclosing operational practices such as congestion management and application-specific traffic engineering.

Lehr et al.’s suggestions include creating a D&T Coordinator (a kind of meta-tool) and advocating the use of an independent third-party measurement infrastructure. They also refer to examples of voluntary transparency reports by Google^7^ and Automattic.^8^ They also attempted to set up an independent measurement platform that can provide new disclosure capabilities, but unfortunately the project seems to have been discontinued and no record of its results were found by us. Also the D&T Coordinator was only presented as a conceptual model and, as far as we could tell, was not implemented.

While our vision bears similarities with the objectives of the D&T Coordinator, the key differences is that we follow a more distributed approach towards D&T that is more fleshed out as well (e.g., because we propose key components and provide a set of starting points).

### Transparency vs. Anonymity

The need to balance the respect for the privacy of Internet users and the desire to have increased transparency into the operations of the network is a tussle that has been studied the past years. The work of [75] was one of the first to describe the importance of Internet transparency and possible approaches towards realizing it without sacrificing (too much) anonymity. They were particularly focused on addressing the relation between transparency and net-neutrality, and their conclusion was that the focus should move from the latter (neutrality) to the former (transparency) as this would ultimately benefit users. This has been followed by a number of proposals all centered around privacy-preserving data collection in networks. For example, [76] have recently proposed an algorithm to provide aggregated insight into network flows, even in settings with limited number of flows.

We address this dichotomy in our work too, but we do go beyond the traditional flow-based approach. For example, for NIP-RQ7 (“how to balance transparency and security?”) we will need to look at the effect of transparency on all Internet actors, including users (e.g., grid operators and citizens) and network operators. We also move beyond the current solutions running on traditional hardware because we exploit network programmability, for instance to address NCP-RQ1 (“What open telemetry measurements are useful for network descriptions?”). The additional telemetry we have access to provides us with metrics that enable the right ratio of openness and protection.

### Defining the Internet

Lehr et al. [25] posit that how to define “the Internet” is not easily answered and rather that it should be viewed from the following different perspectives: (1) the architectural building blocks, (2) the enterprises that use that architecture to offer services, and (3) the customer experience. To illustrate the point that a single definition of the Internet should not be pursued, they provide several thought-provoking examples related to (policies for) an open Internet. For example, security problems are not solely caused by weaknesses in the Internet architecture, but often arise from the applications used. As such, securing the Internet is a shared responsibility.

The three perspectives and examples put forth in this paper illustrate that answering research question POL-RQ2 (“How do we ensure that a responsible Internet represents the interests of the public and the digital society?”) is not trivial and requires balancing the interests of many actors.

### Internet Knowledge Plane

Clark et al. [36] describe their vision of a Knowledge Plane (KP), a globally distributed system that extends the Internet with advanced network management capabilities. The goal of the KP is “a network that can configure itself, that can explain itself, that can repair itself, and does not confound the user with mysteries”. The KP accomplishes this through AI techniques that automatically decide how to configure different parts of the network based on measurements from multiple vantage points, which may be conflicting or incomplete.

The similarity with our work is that a responsible Internet is a global extension of the Internet as well, with the NIP also measuring the network from multiple vantage points like the KP. Another similarity is that the KP supports accountability through an abstract “why” command (returning why something broke in the network) and controllability through a “fix” command (repairs faults in the network).

The main differences with our work are that we focus on providing higher levels of trust and sovereignty for users rather than on automating network management. Also, in our vision (and that of responsible AI), the KP and the network would have to be designed in a transparent and accountable way, which the KP does not consider. Our proposal is furthermore based on open networking, which the KP does not consider.

### SpoVNet

Measurements have been used to verify, validate, and improve the functionality of the Internet since its earliest days. Some projects, however, have taken the idea considerably further. The SpoVNet project [77] developed the notion of application-specific overlay networks, where applications would communicate over network paths that were specifically designed and created according to their performance and security needs. An important component was a measurement framework that would run on every participating node and could be invoked by any SpoVNet application to optimize the overlay [78].

Our NCP shares the aspect of controllability as it provides for adjusting parameters and settings for optimized traffic flows. The responsible Internet is not restricted to using overlay networks to achieve this, however. It is mostly agnostic to the Internet’s current or future architecture and enables controllability for any of them. We naturally share the idea of using ongoing measurements. However, in a responsible Internet, they are not only used for optimization but also as a tool to validate information stored in the NIP.

### SCION

SCION provides the user with control over the paths that their network traffic takes, on an AS-level [9]. In order to achieve this the user is provided with different paths to the desired destination, if available. This gives transparency of the possible paths and the topology of the ASes, not only to the user but also to the network operators.

SCION’s path transparency and control are excellent building blocks within our proposal for a responsible Internet, as it can provide both input for the network descriptions, through the discovered topology, and enables control over how traffic flows through the Internet based on the analysis of the network descriptions.

The key difference with our work is that our approach is more generic: (1) our network descriptions capture a wide range of security-related attributes at the level of an entire network operator and not just of a specific flows and (2) we enable any user to verify these descriptions, not just the entities on a communications path.

### OKN-KISMET

The OKN-KISMET project aims to prototype a knowledge network to improve the security and functioning of three key Internet core systems (naming, addressing, and routing) [79]. In particular, the project aims for a reduction and mitigation of abuses of IP address space, routing, and DNS operations. Their plan is to gather data that can help inform decisions to the end of improved Internet security.

The similarity with our work is that our motivation for the network descriptions has similarities with theirs: they also observe that many potentially useful data sources on the structure of the Internet exist, at various levels of abstraction, but that it remains difficult to extract meaningful information from them to gain knowledge on the structure and evolution of the Internet and to inform policy.

OKN/KISMET focuses on security. It aligns with our work on the NIP in terms of conceptualizing and representing measurements.

## Conclusions

A responsible Internet takes the Internet into the 2020s because it fulfills the widely supported demand for higher levels of trust and sovereignty for critical infrastructure operators and other types of Internet users. We expect this demand to only increase in the near future as economies and societies are moving online at a further accelerated pace, for instance as a result of the Covid-19 pandemic.

Realizing a responsible Internet is an ambitious undertaking with a wide range of challenges lying ahead, as we have illustrated in this paper. However, we believe it is an attainable goal because several building blocks already exist (e.g., various measurement systems) that can be used as a stepping-stone for the development of a responsible Internet’s main components (NIP, NCP, and policies). Also, we expect further thrusts from the close collaboration of practitioners and researchers from different disciplines (e.g., measurements, open networking, and policy development) and from the lessons learned in other complementary areas of technology where the relevance of topics such as trust, sovereignty, and transparency is increasing as well (e.g., cloud services and AI).

While a responsible Internet will put Internet users such as critical infrastructure operators, policy makers, and individuals in the driver’s seat, it will also require them to think differently about the Internet: no longer as a black box, but as a crucial piece of machinery that everyone’s daily life depends on and that we therefore need to have some level of insight in and control over.

We are looking forward to a wider dialogue with the community to make a responsible Internet the new global communications vehicle of the future.

## Footnotes

1 Three of the authors of this paper are affiliated with the ccTLD operator of the Netherlands, .nl.

2 https://maddvipr.org/.

3 https://www.linuxfoundation.org/projects/networking/.

4 https://www.opencompute.org/.

5 http://www.2stic.nl.

6 https://www.fcc.gov/restoring-internet-freedom.

7 https://transparencyreport.google.com/government-removals/overview.

8 https://transparency.automattic.com.
